# 6th Golden Helix Pharmacogenomics Day: pharmacogenomics and individualized therapy

**DOI:** 10.1186/1479-7364-6-19

**Published:** 2012-09-25

**Authors:** Maja Stojiljkovic, Amira Fazlagic, Lidija Dokmanovic-Krivokapic, Gordana Nikcevic, George P Patrinos, Sonja Pavlovic, Branka Zukic

**Affiliations:** 1 Institute of Molecular Genetics and Genetic Engineering, Faculty of Medicine, University of Belgrade, Vojvode Stepe 444a, Belgrade, 11010, Republic of Serbia; 2 National Association for Enhancement and Development of Regenerative Medicine, Belgrade, 11000, Republic of Serbia; 3 University Children’s Hospital, University of Belgrade, Belgrade, 11000, Republic of Serbia; 4 Department of Pharmacy School of Health Sciences, University of Patras, Patras, 26504, Greece

**Keywords:** Pharmacogenomics, educational activity, personalized medicine

## Abstract

The Golden Helix Pharmacogenomics Days are international scientific meetings aiming to educate healthcare professionals and biomedical scientists about pharmacogenomics and personalized medicine. In this meeting report, we provide an overview of the scientific lectures and the topics discussed during the 6th Golden Helix Pharmacogenomics Day that was held in Belgrade, Serbia last June 5, 2012. The scientific program included lectures by the local and international speakers from Europe and the United States.

## The Golden Helix Pharmacogenomics Days

Pharmacogenomics is referred to as the study of variations in DNA sequence and gene expression as related to drug efficacy and toxicity. It is a base for the implementation of personalized medicine, a young but rapidly advancing field of health care. The goal of personalized medicine is to identify genomic and clinical information in order to predict individual risk of developing disease, the course of disease and response to treatment of a person.

The Golden Helix Pharmacogenomics Days are international educational scientific meetings that are organized by the Golden Helix Institute of Biomedical Research [1,2] jointly with the local academic institutions in major cities with large academic hospitals. The aim of these meetings is to provide timely updates on the field of pharmacogenomics and personalized medicine to the local biomedical scientists, healthcare providers and biomedical students in order to educate and inform them on the application of pharmacogenomics in modern medical practice and to bring together faculty members from universities and research institutes from the local scientific arena working in the field of pharmacogenomics in order to initiate collaborative projects in this field to the benefit of the society. Previous Golden Helix Pharmacogenomics Days have been organized in Athens (Greece: May 7, 2009), Thessaloniki (Greece: April 15, 2010) and Alexandroupolis (Greece: April 8, 2011), Cagliari (Italy: October 8, 2011) and Msida (Malta: December 3, 2011) [3].

The 6th Golden Helix Pharmacogenomics Day was organized in Belgrade, Serbia by the Golden Helix Institute of Biomedical Research, the Institute of Molecular Genetics and Genetic Engineering, University of Belgrade, and the National Association for Enhancement and Development of Regenerative Medicine, Belgrade, Republic of Serbia, endorsed by the Pharmacogenomics for Every Nation Initiative (PGENI) and Ministry of Education and Science, Republic of Serbia, and supported by the Complete Genomics, Cryo-Save, Imel Group and Illumina.

The meeting was attended by over 180 registered participants. In this meeting report, we provide an overview of the scientific lectures and present the highlights of this meeting.

## The 6th Golden Helix Pharmacogenomics Day

The most powerful tools of disease research and translational medicine are the next-generation sequencing methods. As a result of their development, we also entered into the golden age of pharmacogenomics. Dr Radoje Drmanac (chief scientific officer, Complete Genomics, Mountain View, CA, USA) delivered the keynote lecture and gave the inspirational overview of sequencing by hybridization, which led to the industrialization of whole genome sequencing. Drmanac first got the idea how to revolutionize sequencing method while he was working at the Genetic Engineering Center in Belgrade (now Institute of Molecular Genetics and Genetic Engineering, University of Belgrade). While Complete Genomics currently has the capacity to sequence 1000 genomes per month, their next big goal is to sequence 100 times more genomes during the same time and with an improved quality, and most importantly, lower costs [4,5]. The whole genome sequencing (WGS) is the ultimate genetic test which is taken only once and used throughout lifetime. It has a diagnostic and predictive values and it will become essential in disease prevention and personalized treatment leading to a longer healthy life [6].

Snezana Pajovic (University of Nis, Faculty of Medicine, Serbia) addressed the importance of pharmacogenomic polymorphisms from the medical point of view. Modern medicine recognizes inter-individual differences in the capability of persons to metabolize a given drug. Contrary to transient adverse drug effects, e.g. caused by allergies, if a person has a genetic disability to metabolize a drug, it will be persistent through lifetime. Pajovic stressed the importance of performing pharmacogenomic tests for the benefit of the patient and as a paradigm of personalized medicine. Furthermore, physicians could easily make a drug choice which is especially important when a life-long therapy is needed. At the end, she pointed out that the pharmacogenomic testing should be used to reduce the overall healthcare costs.

Sonja Pavlovic (University of Belgrade, Institute of Molecular Genetics and Genetic Engineering, Serbia) pointed out that the physicians growingly recognize the need to perform genetic tests in order to optimize the treatment dose. Giving the state of the art of pharmacogenomic testing, Pavlovic highlighted that there is an immense number of candidates for pharmacogenomic testing [7] and that professionals could be confused what genetic variations should be taken into account. Therefore, Pavlovic identified obligatory and recommended pharmacogenomic markers (approved by FDA, EMA, and PMDA) [8,9]. The most relevant pharmacogenomic tests are routinely performed in Serbian accredited laboratories. Unfortunately, the main obstacle of broader use of these tests is that patients have to pay the testing themselves. At the end, Pavlovic reviewed the state of pharmacogenomics in Serbia saying that there is a solid biomedical and molecular-genetic research base that has already been followed by clinical studies and has been implemented in the clinical practice. This progress is mainly accomplished through research projects funded by Ministry of Education and Science of Republic of Serbia, which also endorsed this event.

Vita Dolzan (University of Ljubljana, Faculty of Medicine, Institute of Biochemistry, Slovenia) and Sabina Semiz (University of Sarajevo, Faculty of Pharmacy, Bosnia and Herzegovina) gave valuable insights into the pharmacogenomics in Slovenia and Bosnia and Herzegovina. They presented centers that perform pharmacogenomic tests and have infrastructure for pharmacogenomic research in these countries. Dolzan also presented recently organized interdisciplinary doctoral program in biomedicine as a base for education of future pharmacogenomic experts in Slovenia. Both Dolzan and Semiz are participating, on behalf of their countries, in the PGENI that is coordinated in Europe by the Golden Helix Institute of Biomedical Research [10,11].

George P Patrinos (University of Patras, Patras, Greece) outlined the steps that can be taken by countries with good health system infrastructure, but there are insufficient resources for individual patient genotyping in routine practice to improve patient treatment and reduce healthcare cost. Patrinos underlined that Serbia is one of the 18 European countries that are currently participating in the PGENI, which also endorsed this event [10]. On this occasion, Patrinos revealed preliminary data of pharmacogenomic analysis of 50 Serbian healthy volunteers which will be used in the future to modulate pharmacogenomic practice in Serbia. These results pointed out to some very interesting differences between common pharmacogenomic marker allele frequencies in the Caucasians and Serbian population, further supporting the importance of PGENI to rationalize drug use in developing countries [11].

Florian Graedler (Illumina, Munich, Germany) gave an overview of the next generation sequencing and array technologies and their applications in pharmacogenomics. Serbian experience of pharmacogenetics in clinical practice was presented by Lidija Krivokapic-Dokmanovic (University of Belgrade, University Children’s Hospital, Serbia). A thiopurine drug 6-mercaptopurine is used in maintenance phase of the standard therapy in childhood ALL. In the retrospective and prospective study of thiopurine toxicity in Serbian childhood ALL patients, Dokmanovic explained how the modified therapeutic protocol for treatment of the ALL children was introduced at the University Children’s Hospital in Serbia [12]. In the prospective study, a dose of thiopurine drugs was modified according to the patient’s *TPMT* genotype. These patients, even heterozygous TPMT carriers, had no hematological complications, no febrile neutropenias, and no therapy interruptions [13]. This novel modification of standard therapeutic protocol has significantly improved childhood ALL treatment in Serbia.

Staying focused on the same drug, Branka Zukic (University of Belgrade, Institute of Molecular Genetics and Genetic Engineering, Serbia) gave an overview of the current research on a novel potential pharmacogenomic marker, the variable number of tandem repeats (VNTR alleles), in the promoter region of the *TPMT* gene. The distribution and frequency of VNTR alleles and VNTR genotype of Serbian population were presented. The transcriptional potential of each VNTR allele detected was determined in functional CAT assays [14]. Also, the hypothesis for the role of VNTR region in regulation of *TPMT* gene transcription was presented. Zukic further discussed the latest experiments on thiopurine influence on VNTR region. Decrease of *TPMT* gene promoter activity under the influence of thiopurine drugs was VNTR architecture-dependent [15]. Furthermore, five VNTR alleles are pointed out that display extreme susceptibility for thiopurine therapy. She concluded that preliminary results of the clinical studies confirmed that VNTR genotype could be a novel pharmacogenetic marker which can be truly used for personalizing thiopurine therapy.

Next, Maja Stojiljkovic (University of Belgrade, Institute of Molecular Genetics and Genetic Engineering, Serbia) pointed out that the most frequent variant of uridine diphosphate glucoronosyltransferase 1A1 (*UGT1A1*) gene, (TA)_7_TAA, represents one of the clinically most relevant pharmacogenomic markers. Moreover, this variant is equally important as a diagnostic marker for patients with Gilbert’s syndrome. At this symposium, Stojiljkovic presented the first data about variants in the *UGT1A1* gene found in Serbian Gilbert’s syndrome patients and healthy volunteers. Not only that patients with Gilbert’s syndrome were genotyped so that their hyperbilirubinemia is not confused with more severe hepatic lesions, but also the data deduced from healthy volunteers proved that (TA)_7_TAA pharmacogenomic marker is very important in Serbian population. Stojiljkovic also underlined the third but not least application of the *UGT1A1* genetic test on (TA)_7_TAA. She reminded that this test should be used in clinical trials with investigational drugs to differentiate between persons not knowing to have Gilbert’s syndrome and those in whom hyperbilirubinemia is caused by liver toxicity of a tested drug.

Thromboembolism is a significant cause of morbidity and mortality in adults, and giving that its treatment relies on anticoagulants, adverse effect of the treatment itself could be severe and even lethal. Ljiljana Rakicevic (University of Belgrade, Institute of Molecular Genetics and Genetic Engineering, Serbia) focused her lecture on fine optimization of the drug dose based on pharmacogenomic tests. Giving that anticoagulant drugs have narrow therapeutic range, she highlighted that besides information about genomic variants, attention must be paid to a drug’s interaction with other drugs and the existence of other health conditions and diseases. Rakicevic presented Serbian experience with significant markers for thromboembolism prevention and treatment [16]. These pharmacogenomic markers are one of the many examples of significant inter-ethnical differences that provide important point of view for implementation of pharmacogenetic testing in different populations.

An added value of this symposium was its interdisciplinarity. Side by side with biomedical experts, Hajrija Mujovic (Institute of Social Sciences, Serbia) shared her view on the legal issues of pharmacogenomic testing in Serbia. She defined that the increasing development of biomedicine led to formation of a new legal discipline called medical rights. Mujovic pointed out that principal legal issues of the medical rights are based on social justification and responsibility, health improvement, equality in treatment, non-discrimination, freedom of choice, respect of individual dignity and integrity, personal security and respect of privacy. She added that informed consent is always a prerequisite for genetic testing and that a person should understand what for their sample is going to be used. Furthermore, one must not feel obligated to perform any genetic test because of the fear that otherwise he/she would not be treated properly. Mujovic concluded the talk with a reminder that research practice in general, and thus pharmacogenomic testing as well, should be conducted with respect to the Universal Declaration on Bioethics and Human Rights and International Declaration on Human Genetic Data, both issued by UNESCO [17,18].

The 6th Golden Helix Pharmacogenomics Day in Belgrade provided information on research and application of pharmacogenomics in Serbia and neighboring countries. The most recent data on common clinically applicable pharmacogenomic markers were presented. Also, the importance of WGS was emphasized. WGS is of particular value when it comes to rare pharmacogenomic variants, those that, due to their low frequency, are not screened with usual pharmacogenomic tests, and therefore, cause severe adverse drug effect.

## Conclusions

As with the previous meetings of this international series, the 6th Golden Helix Pharmacogenomics Day highlighted various areas of interest in pharmacogenomics, where many internationally renowned scientists gave excellent overviews of the existing applications and demonstrated the way and the rapid pace that the field is moving. The Golden Helix Institute of Biomedical Research aims to establish the Golden Helix Pharmacogenomics Days as one of the main educational and outreach activities for PGENI [10,11] in European countries, such that healthcare professionals and biomedical scientists are aware of the benefits of this promising new discipline so that the adoption of pharmacogenomics in the mainstream clinical practice is expedited, particularly in developing countries. Reciprocally, the spread of pharmacogenomic applications will significantly benefit the general public in these countries. The final message to take home is that education of general public and professionals on the pharmacogenomics is essential for the success of personalized medicine.

## Competing interests

The authors declare that they have no competing interests.

## Authors' contributions

MS, AF, LDK, GN, GPP, SP and BZ participated in the design and preparation of this meeting report. All authors read and approved the final manuscript.
